# Identifying shared genetic loci and common risk genes of rheumatoid arthritis associated with three autoimmune diseases based on large-scale cross-trait genome-wide association studies

**DOI:** 10.3389/fimmu.2023.1160397

**Published:** 2023-06-12

**Authors:** Ya-Ping Wen, Zu-Guo Yu

**Affiliations:** ^1^ National Center for Applied Mathematics in Hunan, Xiangtan University, Hunan, China; ^2^ Key Laboratory of Intelligent Computing and Information Processing of Ministry of Education, Xiangtan University, Hunan, China

**Keywords:** rheumatoid arthritis, autoimmune diseases, association studies, shared genes, cross-trait

## Abstract

**Introduction:**

Substantial links between autoimmune diseases have been shown by an increasing number of studies, and one hypothesis for this comorbidity is that there is a common genetic cause.

**Methods:**

In this paper, a large-scale cross-trait Genome-wide Association Studies (GWAS) was conducted to investigate the genetic overlap among rheumatoid arthritis, multiple sclerosis, inflammatory bowel disease and type 1 diabetes.

**Results and discussion:**

Through the local genetic correlation analysis, 2 regions with locally significant genetic associations between rheumatoid arthritis and multiple sclerosis, and 4 regions with locally significant genetic associations between rheumatoid arthritis and type 1 diabetes were discovered. By cross-trait meta-analysis, 58 independent loci associated with rheumatoid arthritis and multiple sclerosis, 86 independent loci associated with rheumatoid arthritis and inflammatory bowel disease, and 107 independent loci associated with rheumatoid arthritis and type 1 diabetes were identified with genome-wide significance. In addition, 82 common risk genes were found through genetic identification. Based on gene set enrichment analysis, it was found that shared genes are enriched in exposed dermal system, calf, musculoskeletal, subcutaneous fat, thyroid and other tissues, and are also significantly enriched in 35 biological pathways. To verify the association between diseases, Mendelian randomized analysis was performed, which shows possible causal associations between rheumatoid arthritis and multiple sclerosis, and between rheumatoid arthritis and type 1 diabetes. The common genetic structure of rheumatoid arthritis, multiple sclerosis, inflammatory bowel disease and type 1 diabetes was explored by these studies, and it is believed that this important discovery will lead to new ideas for clinical treatment.

## 1 Introduction

It is well known that the major function of the immune system is to protect the host from environmental agents such as microbes or chemicals, thereby preserving the integrity of the body (1). When the body is injured or invaded by pathogenic microorganisms, acute inflammatory reaction is often accompanied, and the immune system and inflammatory mechanism are inseparable (2). However, uncontrolled inflammatory and immune responses can lead to immune system disorders that trigger autoimmune diseases, such as rheumatoid arthritis (RA), inflammatory bowel disease (IBD) and type 1 diabetes mellitus (T1D) (3). Autoimmune diseases are complex diseases caused by genetic and environmental factors (4). The clinical manifestations of these diseases are familial clustering, and multiple immune diseases can occur simultaneously in the same individual, which indicates that autoimmune diseases have a common genetic background. Moreover, genomic studies have shown that the same gene loci can be found in various autoimmune diseases, and genetic overlap exists in autoimmunity, indicating that autoimmune diseases may have the same molecular mechanism (5, 6).

RA is a chronic, inflammatory autoimmune disease that can cause severe movement impairment and deterioration of quality of life (7). In twin and familial studies, the overall heritability of rheumatoid arthritis is estimated to be about 50%-65% (8). It’s suggested that rheumatoid arthritis is familial, individuals with a family history of rheumatoid arthritis are at increased risk of developing rheumatoid arthritis due to common genetic factors (9–11). Multiple sclerosis (MS) is an inflammatory autoimmune disease in which the myelin sheath and spinal cord in the central nervous system are damaged, which can result in demyelination and axonal loss. Some studies have suggested that patients with multiple sclerosis have an increased risk of rheumatoid arthritis (12–14). IBD is a chronic non-specific inflammatory condition of the gastrointestinal tract. In recent years, studies have found that the gene predictive risk of RA is positively correlated with the increased risk of IBD (15, 16). Yang et al. studied the common genetic structure of MS and IBD through large-scale genome-wide association studies (GWAS), and the results showed that the comorbidity of MS and IBD has a biological basis (17). In addition, previous studies have shown that individuals with RA, MS or IBD have an increased risk of influenza and related complications (18), and an increased risk of depression, especially in women compared to men (19). Diabetes mellitus (DM) is a chronic disease that causes hyperglycemia due to defective insulin secretion or impaired biological action. DM is a group of physiological dysfunctions characterized by hyperglycemia resulting directly from insulin resistance, inadequate insulin secretion, or excessive glucagon secretion (20). Type 1 diabetes (T1D) is an endocrine disorder in which pancreatic β cells stop producing insulin, typically due to autoimmune destruction (21). Studies have shown that RA is associated with abnormal glucose metabolism, which may lead to the development of DM (22, 23), and patients with MS may increase the risk of developing T1D (24). Ahmad and Ahsan have revealed common risk genes of RA and MS, MS and T1D through reported familial and genetic links (25). Andersen et al. have shown that when parents have RA, IBD, or DM, offspring are at increased risk (26). Recently, Zhao et al. collected summary statistics from GWAS about seven autoimmune diseases, including celiac disease (CEL), MS, primary biliary cirrhosis (PBC), RA, ulcerative colitis (UC), SLE, and T1D to analyze genetic associations (27). Although there is an epidemiological association between RA, MS, IBD and T1D, whether this reflects a common genetic etiology is unclear. Therefore, the purpose of this paper is to reveal the genetic relationships of RA, MS, IBD, and T1D through large-scale cross-trait GWAS analysis.

GWAS combining multiple diseases have become useful tools to identify risk locus for autoimmune diseases, genetic variant associated with multiple diseases, and biological pathways associated with diseases (28–31). Based on the hypothesis that there is a common genetic cause between autoimmune diseases, in this study, we use GWAS summary statistics to investigate the shared genetic capacity of RA, MS, IBD and T1D at the individual variation level and at the genome-wide level, respectively. Firstly, the genetic relationships between RA and MS, RA and IBD, RA and T1D are analyzed. The global genetic associations among diseases are analyzed by linkage disequilibrium score regression (LDSC), and the local genetic associations among diseases are estimated by using ρ−HESS. Then cross-trait meta-analysis is used to identify the shared genetic components between RA and MS, RA and IBD, RA and T1D. Genome-wide association analysis and transcriptome association studies are used to identify potentially the common risk genes among RA, MS, IBD and T1D. Finally, Mendelian randomization is used to analyze the causal relationship between RA and MS, RA and IBD, and RA and T1D respectively. In summary, we leverage large-scale GWAS summary statistics data and preceding genetic methods to gain insight into mechanistic links among RA, MS, IBD and T1D. Our purpose is to identify the common risk genes among RA, MS, IBD and T1D, and provide biological interpretation for common risk genes.

## 2 Materials and methods

### 2.1 Datasets

For summary statistics from GWAS about rheumatoid arthritis (RA), multiple sclerosis (MS), inflammatory bowel disease (IBD), the GWAS summary-level data are downloaded from a publicly accessible database GeneATLAS (32). Specifically, the RA meta-analysis summary statistics include 5082 cases and 447182 controls, MS meta-analysis summary statistics include 1406 cases and 450858 controls and IBD meta-analysis summary statistics combine 3878 cases and 448386 controls. The total 452264 samples are all European-ancestry individuals from UK Biobank, and we used 623944 genotype variants that passed quality control. Summary statistics about type 1 diabetes (T1D) (PMID: GCST90013791) which was uploaded on 02/22/2021 (33) were downloaded from the database NHGRI-EBI GWAS Catalog (34). The T1D meta-analysis summary statistics include 181,214 individuals of European ancestry with 6,294,828 genotype variants. The numbers for cases and controls are not provided in the NHGRI-EBI GWAS Catalog database, but we need not to use this kind of information in our study.

### 2.2 Methods

#### 2.2.1 LD score regression analysis

To evaluate the genetic correlation between RA and MS, RA and IBD, RA and T1D, the linkage disequilibrium score regression (LDSC) (35) was applied to assess the genetic correlation r_g_ between RA and MS, IBD, T1D. We applied LDSC to estimate SNP heritability and LD-score intercept for RA, MS, IBD and T1D, respectively. European-ancestry population of 1000 Genome Project Phase 3 (36) was used as reference panel, from which 1.2 million SNPS were obtained for pre-calculated LD-scores.

#### 2.2.2 Local genetic correlations analysis

To investigate whether there are local genetic correlations between RA and MS, RA and IBD, RA and T1D, we estimated the local genetic correlations between each pair of traits in pre-specified LD independent segments using ρ−HESS (37). The LD-independent blocks are used to calculate local heritability and genetic covariance. However, when we calculated the local genetic correlation using 623,944 SNPs (RA, MS, IBD), we found that there are empty loci on chromosome 1 (chr1:178944309-178954470) and chromosome 7 (chr7:124156805-124167552) in 1703 pre-designated independent fragments, so we combined these loci with nearby loci. Accounting for Bonferroni correction, if P_ρ−HESS_ < 0.05/1701(2.93×10^−5^), there are significant genetic correlations between RA and MS, RA and IBD. For RA and T1D, in addition to the above two regions, there were three empty locus on chr2:95326452-98995201, chr6:29737971-30798168, chr15:20001200-21131604, and we combined these locus with nearby loci, so the significant threshold is P_ρ-hess_ < 0.05/1698 (2.94 ×10^-5^).

#### 2.2.3 Cross-trait meta-analysis

After estimating the genetic correlations between RA and MS, RA and IBD, RA and T1D, we used R packet cross phenotype association (CPASSOC) (38) to analyze the GWAS cross-trait association. CPASSOC includes Shet (for heterogeneous data) and Shom (for homogenous data). We applied the PLINK (39) clustering function to identify the independent and significant SNPs, and the parameter is set as –cluster-p1 1.6×10^-8^–cluster-p2 1×10^-5^ –cluster-r2 0.2 –cluster-kb 500, indicating that SNPs with a P-value less than 1 × 10^−5^, r^2^ greater than 0.2 and less than 500 kb away from the peak value will be assigned to the cluster of the peak value.

#### 2.2.4 Genome-wide gene-based analysis

In gene-based analysis, genetic variation is annotated, i.e., SNPs correspond to the corresponding gene according to the position on the chromosome, and gene-based association analysis is carried out. The MAGMA (40) analytical model uses multiple linear principal component regression to detect the correlation between genes and the disease. In this study, MAGMA gene analysis is used to identify significant genes associated with RA, MS, IBD and T1D, respectively. Using European-ancestry population of 1000 Genomes Project Phase 3 as reference and Genome Reference Consortium Human Build 37 (hg19) as the SNP locations for gene annotation, we found that 301949 (48.39%) of the total 623944 SNPs are mapped to 17446 genes.

#### 2.2.5 Transcriptome-wide association analysis

To detect genes associated with RA, MS, IBD, and T1D in different tissues, we performed transcriptome-wide association analysis by using e-MAGMA (41). e-MAGMA transforms genome-wide association summary statistics into gene-level statistics by assigning risk variants to its putative genes based on tissue-specific eQTL information. We used eQTL information from 47 tissues of the GTEx (version 8) reference panel (42). In total, we performed TWASs for each trait, one tissue-trait pair at a time.

#### 2.2.6 Enrichment analysis and protein-protein interaction network analysis

In order to understand the underlying biological pathways for the identified shared risk genes in RA with MS, IBD, and T1D, we used the tool Enrichr web server (43) to implement the Kyoto Encyclopedia of Genes and Genomes (KEGG) pathway enrichment analysis. The significant criterion is that the adjusted p-value less than 0.05. In addition, we applied STRING (version 10) (44) to analyze the protein-protein interaction (PPI) network.

#### 2.2.7 Mendelian randomization analysis

We performed MR analysis using MR-PRESSO (45) between RA and MS, RA and IBD, RA and T1D since they are genetically correlated. We built the MR instruments based on LD-independent SNPs.

## 3 Results

### 3.1 Genetic correlation between RA and MS, RA and IBD, RA and T1D

We evaluated the global genetic correlation of RA and MS, RA and IBD, RA and T1D using LD score regression (LDSC). RA has the strongest genetic correlation with MS, with a correlation coefficient of 0.4289, followed by RA and IBD, with a correlation coefficient of 0.3743, and then RA and T1D, with a correlation coefficient of -0.3157 (**Table 1**). Furthermore, the LD score intercepts for RA, MS, IBD and T1D are 0.9982 (Se = 0.0097), 1.0172 (Se = 0.0105), 1.0156 (Se = 0.011) and 0.9933 (Se = 0.0098), respectively, indicating that most of the inflation is due to polygenic effect rather than population structure or sample overlap (46).

**Table 1:** Genetic correlation of RA and MS, RA and IBD, RA and T1D.

Trait 1	Trait 2	r_g_	r_g_ − s_e_	p-value	g_cov_	g_cov_ − s_e_
RA	MS	0.4289	0.2932	0.1434	0.0019	0.0012
	IBD	0.3743	0.1504	0.0128	0.0036	0.0013
	T1D	-0.3157	0.0951	0.0009	-0.0102	0.0034

**Figure 1:**
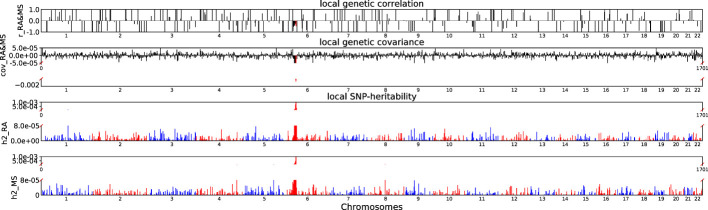
Local genetic correlation and local SNP-heritability between RA and MS. For each sub-figure, the top sub-figure represents local genetic correlation, the second represents local genetic covariance. In these two sub-figures, significant local genetic correlation and covariance after multiple testing correction are highlighted in red. Bottom two sub-figures represent local SNP-heritability for individual trait. The X-axis represents the chromosome, h2_RA represents the SNP-heritability of RA, h2_MS represents the SNP-heritability of MS, r_RA-MS represents the genetic correlation between RA and MS, cov_RA-MS represents the genetic covariance between RA and MS.

**Figure 2:**
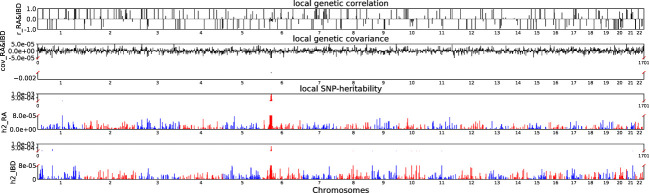
Local genetic correlation and local SNP-heritability between RA and IBD. For each sub-figure, the top sub-figure represents local genetic correlation, the second represents local genetic covariance, bottom two sub-figures represent local SNP-heritability for individual trait. The X-axis represents the chromosome, h2_RA represents the SNP-heritability of RA, h2_IBD represents the SNP-heritability of IBD, r_RA-IBD represents the genetic correlation between RA and IBD, cov_RA-IBD represents the genetic covariance between RA and IBD.

**Figure 3:**
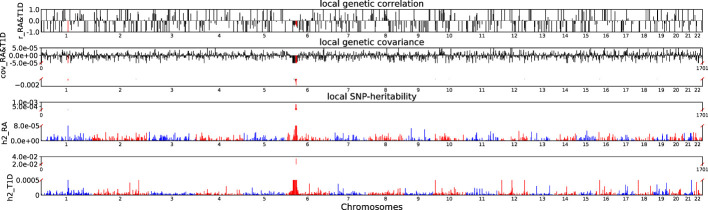
Local genetic correlation and local SNP-heritability between RA and T1D. For each sub-figure, the top sub-figure represents local genetic correlation, the second represents local genetic covariance. In these two sub-figures, significant local genetic correlation and covariance after multiple testing correction are highlighted in red. Bottom two sub-figures represent local SNP-heritability for individual trait. The X-axis represents the chromosome, h2_RA represents the SNP-heritability of RA, h2_T1D represents the SNP-heritability of T1D, r_RA-T1D represents the genetic correlation between RA and T1D, cov_RA-T1D represents the genetic covariance between RA and T1D. In the bottom two subfigures, we did not use the same y-axis as **Figures 1**, **2** because the difference on the order of magnitude for h2_T1D and h2_RA is too large.

Accounting for Bonferroni correction the local genomic regions around individual RA loci from GWAS showed signals of genetic overlap with MS (**Figure 1**). Although RA and MS have positive global genetic correlation using LDSC, we identified two regions (chr6:31571218-32682664, P_ρ−HESS_ = 5.82×10^−17^, and chr6:32682664-33236497, P_ρ−HESS_ = 2.72 × 10^−12^) that show genome wide significant negative local genetic correlation between RA and MS using heritability estimation from summary statistics (ρ−HESS). This reverse result may be caused by the different definitions of SNP heritability and genetic covariance between ρ−HESS and LDSC. We used ρ−HESS method to evaluate local genetic correlations between RA and IBD. There is no significant local genetic correlated regions (**Figure 2**), this means that the genetic association between RA and IBD is likely to be shared genetic variants across the genome rather than strong associations in specific genomic regions. The local genetic correlation between RA and T1D is negative in the chromosome 1 and the chromosome 6 regions (**Figure 3**). There are four significant local genetic correlated regions (chr6: 32682664-33236497, P_ρ−HESS_ = 2.90 × 10^−14^, chr1:113273306-114873845, P_ρ−HESS_ = 1.22 × 10^−11^, chr6: 31571218-32682664, P_ρ−HESS_ = 5.73 × 10^−10^,and chr6: 33236497-35455756, P_ρ−HESS_ = 1.26 × 10^−6^).

### 3.2 Identification of risk SNPs from cross-trait meta-analysis of RA, MS, IBD and T1D

We conducted cross-trait meta-analysis to identify risk SNPs that may share association with RA and MS, RA and IBD, RA and T1D using the Cross Phenotype Association (CPASSOC) package (PCPASSOC < 5×10−8/3(1.6×10−8)).

After excluding SNPs that are genome-wide significant in the respective single-trait GWAS, 58 independent loci reached genome-wide significance for RA and MS, 27 of which have been verified to be significantly related to RA and/or MS by GWAS Catalog database verification (**Supplementary Table S1**). Although most of the independent loci we found were located in the MHC region, we also found loci in the non-MHC region. The loci rs6679677 (on chr1) is close to the PTPN22 gene. The gene involved in regulating CBL function in T cell receptor signaling pathway, and mutations in this gene may be linked to a range of autoimmune diseases rheumatoid arthritis (47). The loci rs7731626 (on chr5) is mapped to ANKRD55 gene which is associated with RA (48) and MS (49).

86 independent loci reached genome-wide significance for RA and IBD, which 46 in this locus have previously been associated to RA and/or IBD (**Supplementary Table S2**). The loci rs3130695 is mapped to including HLA-B and HLA-C genes which from the HLA class I region is associated with RA (13, 50). The locus rs34213882 and rs9263717 are mapped to HLA-C genes. The genes had genome-wide significant association with IBD. The loci rs11465802 (on chr1), rs11209026 and rs3024505 (on chr1) are mapped to IL23R, C1orf141, and IL10 genes associated with IBD (51). In addition, there are loci rs6679677(on chr1) which is mapped to PTPN22 gene associated with RA. The loci rs1801274(on chr1) is mapped to FCGR2A gene which is associated with RA and IBD. The loci rs2076756 (on chr16) is mapped to NOD2 associated with RA.

107 independent loci reach genome-wide significance for RA and T1D, 47 of which have previously been associated to RA and/or T1D (**Supplementary Table S3**). Loci rs2856997, rs2070121, rs7383287 are mapped to HLA-DOB genes which are associated with RA. Loci rs2534674, rs2534671, rs6915833 are mapped to MICB genes which are associated with RA (Ancha et al., 2023). Loci rs1150755, rs12198173 are mapped to APOM genes which are associated with RA. Loci rs2233977, rs20547, rs1063646, rs9263719, rs3094663, rs6916921, rs3819299 and rs12614 are mapped to C6orf15, PSMB9, PSORS1C1, PSORS1C2, NFKBIL1, HLA-B and CFB genes which are associated with RA (52–54). In addition to the above findings in genes located in the MHC region, we also found RA-related loci in non-MHC regions. The loci rs7041847 (on chr9) is mapped to GLIS3, and the loci rs7200786 (on chr16) is mapped to CLEC16A which are associated with RA (55, 56). The loci rs2281808 (on chr20) is mapped to SIRPG, and the rs1805761(on chr12) is mapped to M6PR which are associated with T1D (57, 58). The loci rs6859219 (on chr5) is mapped to ANKRD55 genes which are associated with RA and T1D. Locus rs2847281 and rs7234029 (on chr18) are mapped to PTPN2 genes which are associated with RA and T1D (59, 60). These locus rs705708 (on chr12), rs706778 (on chr10), rs6669008 (on chr1), rs1788103 (on chr18) and rs9976767(on chr21) are mapped to ERBB3, IL2RA, MAGI3, CD226 and UBASH3A which are associated with RA and T1D.

### 3.3 Genes identified by genome-wide and transcriptome-wide studies

**Figure 4:**
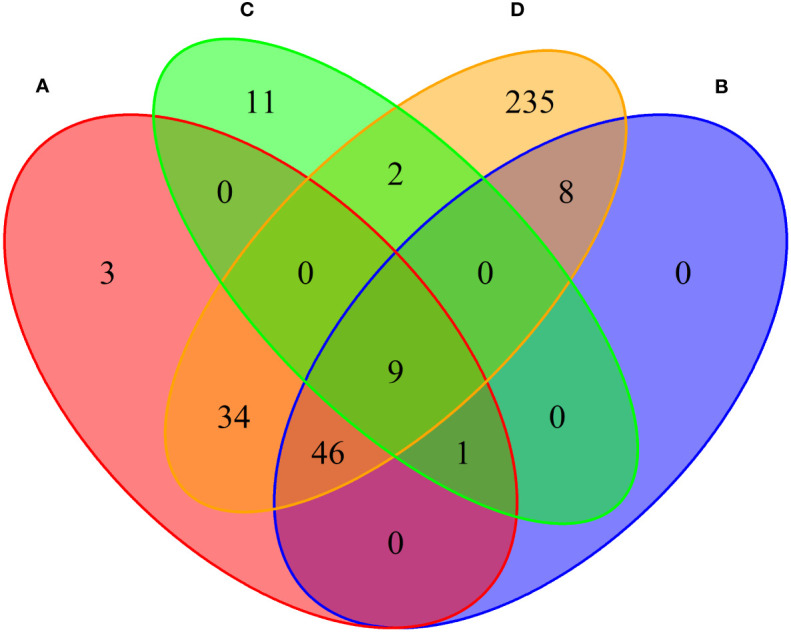
Venn diagram of the number of genes identified by MAGMA method. **(A)** RA associated genes, **(B)** MS associated genes, **(C)** IBD associated genes, **(D)** T1D associated genes.

We conducted MAGMA genome-wide gene-based analysis to identify genes associated with RA, MS, IBD, and T1D, respectively. The numbers of genes identified are shown in **Figure 4**. It can be seen from the figure that after Bonferroni correction of the total 19427 genes, 93 genes (P_MAGMA_< 2.87×10^−6^) are identified as significantly correlated with RA; 64 genes are related to MS; 23 genes are associated with IBD; 334 genes are related to T1D (**Supplementary Table S4**). There are 56 overlapping genes between RA and MS; 10 overlapping genes between RA and IBD; 89 overlapping genes RA and T1D; 10 overlapping genes RA, MS and IBD; 55 overlapping genes RA, MS and T1D; 9 overlapping genes RA, IBD and T1D; 9 among the four diseases (**Table 2**).

**Table 2:** Shared risk genes for RA, MS, IBD and T1D in MAGMA analysis.

Gene	Position	No. SNPs	P_MAGMA_			
			RA	MS	IBD	T1D
BTNL2	Chr6:32362513-32374900	39	8.77e-49	1.08e-22	4.79e-11	5.06e-90
HLA-DRA	Chr6:32407619-32412823	51	7.42e-39	1.60e-40	3.52e-8	6.01e-82
ATF6B	Chr6:32083045-32096017	16	5.10e-34	1.72e-13	8.85e-8	7.91e-80
EHMT2	Chr6:31847536-31865464	20	1.96e-32	9.18e-24	1.05e-9	5.18e-61
HLA-DQB1	Chr6:32627241-32634466	12	2.89e-32	8.09e-14	5.67e-12	1.09e-44
TAP2	Chr6:32789610-31865464	48	3.03e-22	1.19e-12	1.71e-08	1.63e-121
TRIM31	Chr6:30070674-30080867	31	2.01e-08	1.02e-7	7.90e-10	2.01e-52
NELFE	Chr6:31919864-31926864	14	1.96e-22	1.34e-14	5.19e-10	1.05e-68
MICA	Chr6:31367561-31383090	38	3.11e-13	1.07e-7	1.23e-6	8.61e-145

**Figure 5:**
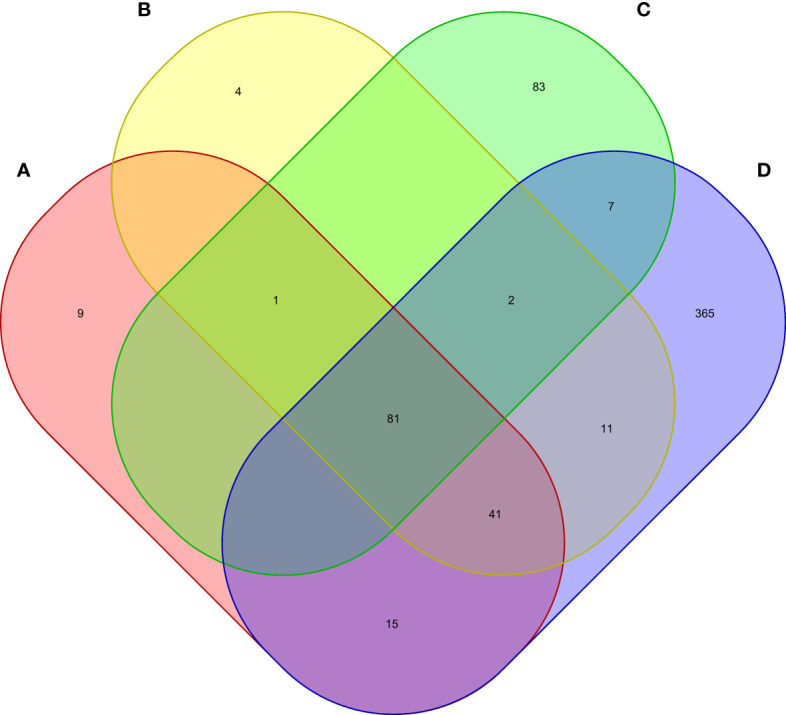
Venn diagram of the number of genes identified by e-MAGMA method. **(A)** RA associated genes, **(B)** MS associated genes, **(C)** IBD associated genes, **(D)** T1D associated genes.

**Figure 6:**
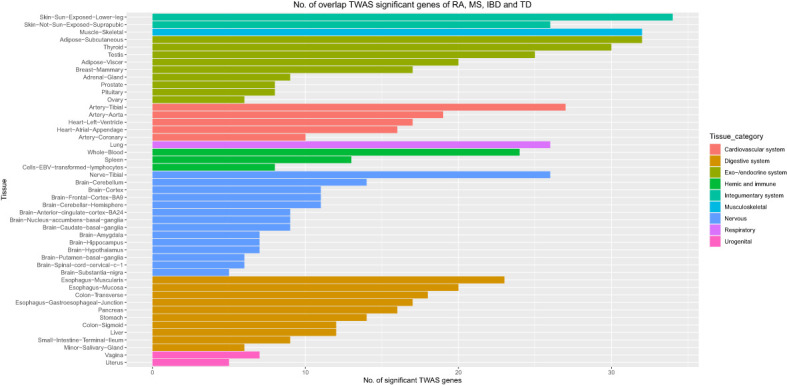
Number of overlap TWAS significant genes of RA, MS, IBD and T1D traits across 47 tissues. The X axis shows the count of genes. The Y axis lists tissues. Colors represent different tissue categories. TWAS, transcriptome-wide association studies, No. number.

Moreover, we carried out eMAGMA transcriptome-wide gene-based analysis with RA, MS, IBD, and T1D, respectively, and the result are shown in **Figure 5**. The genes significantly associated with 47 tissues of each disease are identified successively, 147, 140, 174 and 522 genes significantly associated with RA, MS, IBD and T1D are identified, respectively (**Supplementary Tables S5-S8**). There are 123 overlapping genes between RA and MS; 82 overlapping genes between RA and IBD; 137 overlapping genes RA and T1D; 82 overlapping genes RA, MS and IBD; 122 overlapping genes RA, MS and T1D; 81 overlapping genes RA, IBD and T1D; 81 among the four diseases, eight out of nine common risk genes detected by MAGMA are also detected by e-MAGMA (**Supplementary Table S9**), we identified 82 common risks among the four diseases. 40 of the 82 risk genes are significantly associated with the disease reported in previous studies. We also paid attention to the enrichment analysis of 82 common risk genes in tissues. As shown in **Figure 6**, it was found that 34 risk genes are mainly enriched in integumentary system of skin sun exposed lower leg, 32 are enriched in muscle skeletal, and 32 are enriched in adipose subcutaneous.

### 3.4 KEGG enrichment analysis and PPI network analysis results

**Figure 7:**
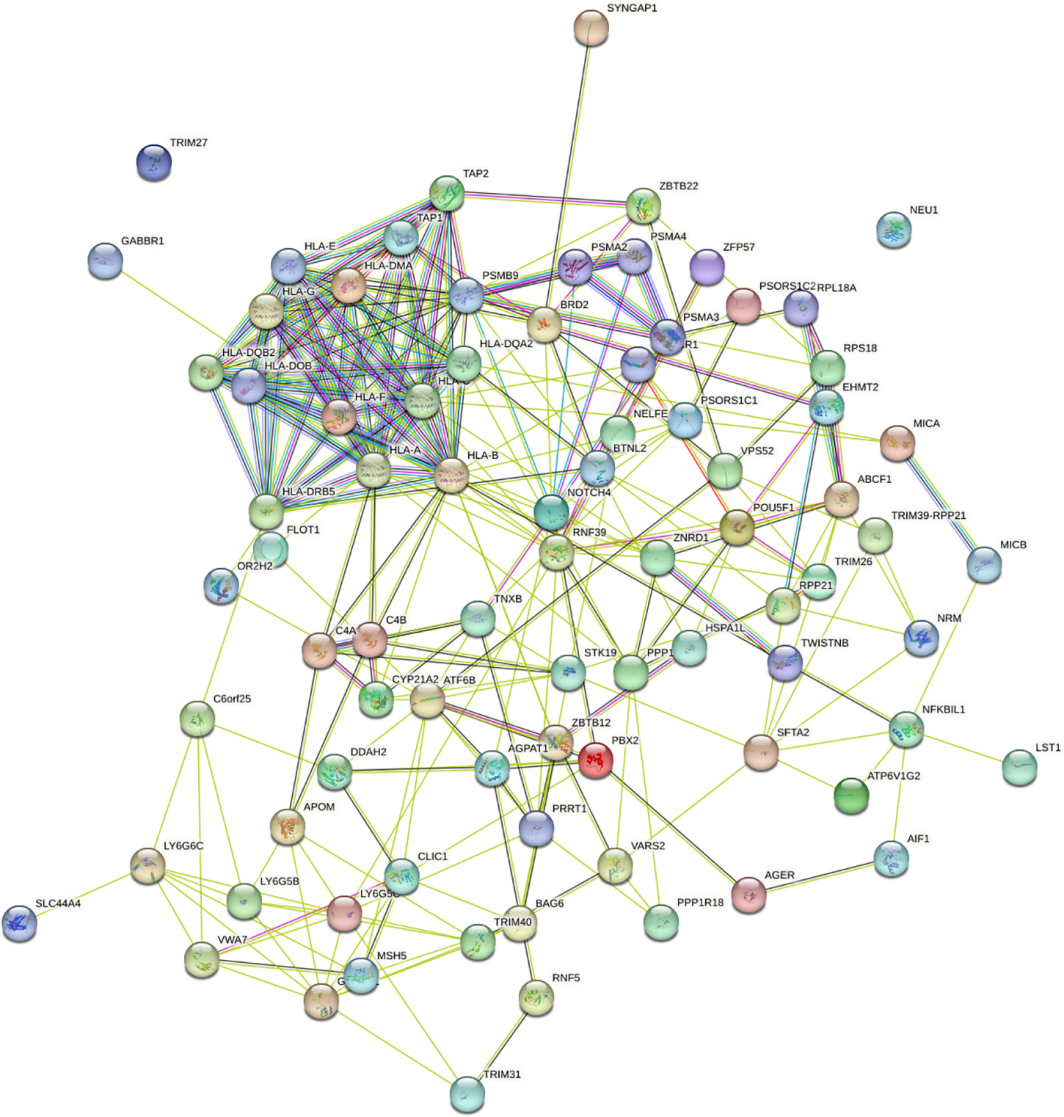
The PPI network of 82 common risk genes.

To understand the impact of risk genes in biological pathways, we used Enrichr to enrich the co-risk genes in a KEGG functional analysis. We found that 35 biological pathways are significantly enriched. As shown in the **Table 3**, the strongest enrichment signal is antigen processing and presentation, which include 13 enriched genes (HLA-DRB5, HSPA1L, HLA-B, TAP2, HLA-C, TAP1, HLA-A, HLA-F, HLA-G, HLA-E, HLA-DMA, HLA-DOB, HLA-DQA2). In order to understand the interaction between common risk genes of four diseases, we used STRING for PPI network analysis. The 82 risk genes have 279 gene pairs interacting in PPI network, with an average clustering coefficient of 0.421, and the composite scores of all the interacting genes is not less than 0.4, among which the score of 58 gene pair composite score were more than 0.95. The five hub genes (degrees > 15) that extensively interact with other genes in the PPI network are HLA-B, HLA-A, HLA-C, PSMB9, HLA-F. The PPI network of common risk genes are shown in the **Figure 7**.

**Table 3:** The KEGG pathway was significantly enriched in 82 common risk genes.

Pathway	No. Genes	Adjusted P-value	Pathway	No. Genes	Adjusted P-value
Antigen processing and presentation	13	6.51e-16	Systemic lupus erythematosus	6	1.07e-04
Allograft rejection	10	1.75e-14	Viral carcinogenesis	7	1.07e-04
Graft-versus-host disease	10	3.50e-14	Rheumatoid arthritis	5	2.04e-04
Type I diabetes mellitus	10	3.50e-14	Cellular senescence	6	2.07e-04
Autoimmune thyroid disease	10	2.75e-13	Intestinal immune network for IgA production	4	2.07e-04
Viral myocarditis	10	5.43e-13	Endocytosis	7	3.52e-04
Phagosome	13	7.43e-13	Toxoplasmosis	5	4.17e-04
Epstein-Barr virus infection	12	4.84e-10	Inflammatory bowel disease	4	6.05e-04
Cell adhesion molecules	10	5.85e-9	Leishmaniasis	4	0.00112
Human T-cell leukemia virus 1 infection	11	1.60e-8	Th1 and Th2 cell differentiation	4	0.00213
Herpes simplex virus 1 infection	13	1.10e-6	Hematopoietic cell lineage	4	0.00271
Human cytomegalovirus infection	9	3.15e-06	Th17 cell differentiation	4	0.00351
Natural killer cell mediated cytotoxicity	7	9.16e-06	Influenza	4	0.01894
Kaposi sarcoma-associated herpesvirus infection	8	9.62e-06	Tuberculosis	6	0.02151
Human immunodeficiency virus 1 infection	8	1.71e-05	Longevity regulating pathway	3	0.02763
Staphylococcus aureus infection	6	1.71e-05	Primary immunodeficiency	2	0.03339
Asthma	4	4.78e-05	ABC transporters	2	0.04484
Human papillomavirus infection	9	5.04e-05			

### 3.5 Instrumental variable analysis

Finally, we used MR-PRESSO instrumental analysis to develop evidence for causality in the relationship between RA and MS, RA and IBD, RA and T1D, and the results are shown in **Table 4**. As shown in the **Table 4**, the finding that there may be a causal relationship between T1D and RA. We found a possible new causal relationship between T1D and RA. Although no relevant studies have confirmed the causal relationship between T1D and RA, the risk of type 1 diabetics developing RA later in life may be attributed in part to the presence of the PTPN22 allele (61).

**Table 4:** Mendelian Randomization analysis between RA, MS, IBD and T1D.

Exposure	Outcome	Causal Estimate	Sd	T-stat	P-value^1^
RA	MS	-0.000756071	0.005449628	-0.138738	0.889728
MS	RA	-0.1195902	0.05367141	-2.228192	0.03170483
RA	IBD	-0.006132929	0.003543663	-1.730675	0.08381547
IBD	RA	0.01654898	0.02420687	0.683648	0.4970677
RA	T1D	-0.7789012	0.5110154	-1.524222	0.1276694
T1D	RA	-307.0967	80.94547	-3.793871	0.0002637433

Another finding is that there may be a causal relationship from MS to RA, but not vice versa, which supports the idea that common immunologic pathways, involving IL-17 and Th17, may be one of the mechanisms through which MS increases susceptibility to RA(17). MS diagnosis increased the likelihood of a patient’s subsequent diagnosis of rheumatoid arthritis. Our MR-PRESSO analysis showed no causal relationship between RA and IBD after adjusting pleiotropy. These results further support our findings that the shared genetic effects between RA and IBD are more likely to be pleiotropic effects, rather than causal etiology or mechanism.

## 4 Discussion

In this study, we aimed to determine the genetic relationships among RA, MS, IBD, and T1D by large-scale cross-trait GWAS analysis. Firstly, LDSC is used to identify the genome-wide genetic relationships between RA and MS, RA and IBD, RA and T1D. We found that there are statistically significant genetic relationships between RA and MS, RA and IBD, RA and T1D. Secondly, ρ−HESS is adopted to identify the local genetic relationships between RA and MS, RA and IBD, and RA and T1D. It was found that there are two significant local genetic correlation regions between RA and MS, and four significant local genetic correlation regions between RA and T1D. Thirdly, the CPASSOE method is used to identify significant correlation loci between RA and MS, RA and IBD, RA and T1D. It was found that there are 58 significant correlation loci between RA and MS, 86 significant correlation loci between RA and IBD, and 107 significant correlation loci between RA and T1D. Fourthly, by using the multiple omics method MAGMA and e-MAGMA to identify the common risk genes for four diseases, we found that 82 risk genes show significant association with all four diseases, and 40 of these diseases have been confirmed to be associated with at least one disease. Fifth, we introduced the biological functions of the 82 risk genes found through tissue and organ enrichment analysis, biological pathway enrichment analysis and protein-protein analysis, and found that 82 common risks genes are mainly concentrated in skin sun exposed lower leg, muscle skeletal, adipose subcutaneous, and 35 biological pathways. Finally, we used the MR-PRESSO method to identify the causal relationship between RA and MS, RA and IBD, RA and T1D, and found that there may be causal relationship between RA and T1D, RA and MS, but there is no causal relationship between RA and IBD. The reason of the genetic relationship between RA and IBD is due to pleiotropy effects.

In this study, 82 common risk genes related to RA, MS, IBD and T1D were identified, among which a large number of common genes were found in the HLA region, which plays an important role in immune response. Immune response is one of the main factors affecting RA, MS, IBD and T1D (12, 51, 62). TSBP1 gene has been reported to be associated with four diseases (63–66). Although CCHCR1 gene has been reported to be associated with MS, IBD and T1D diseases (67–69), it may also be important for RA. Twenty-two genes (FLOT1, VARS2, POU5F1, MICA, MICB, NFKBIL1, TAP2, TAP1, BRD2, TNXB, AGPAT1, TRIM31, APOM, TRIM27, SLC44A4, RNF39, AGPAT1, ABCF1, RNF5, CYP21A2, PSORS1C1, LST1) have been reported to be associated with at least one disease. Although no relevant study shows the correlation between TRIM26 and RA, MS, IBD, T1D, we found that TRIM26 is a member of the TRIM protein family, encoded in the locus of major histocompatibility complex Class I region, and TRIM26 interacts with TAB1 and specifically catalyzes K11-linked polyubiquitination of TAB1, which facilitates TAK1 activation and initiates downstream signaling, and finally positively regulated TLRS-mediated inflammatory cytokines (70). AIF1 is a 17kDa cytoplasmic calcium-binding inflammatory scaffold protein, which is mainly expressed in immune cells. AIF1 promotes the expression of inflammatory mediators such as cytokines, chemokines and inducible nitric oxide synthase (iNOS), promoting the proliferation and migration of inflammatory cells (71). CLIC1 participates in inflammatory processes by regulating macrophage phagosomal functions such as pH and proteolysis, CLIC1 regulates DC phagosomal pH to ensure optimal processing of antigen for presentation to antigen-specifific T-cells (72).

There are also some limitations in this paper. Firstly, Bonferroni correction is the most stringent multiple testing correction method. In genome-wide association analysis, in order to control the probability of false positives, the threshold is often adjusted with Bonferroni correction. However, due to linkage disequilibrium between GWAS variants, there may be cases where multiple variants or SNPs are linked to each other, so it is not entirely correct to assume that each association test of a GWAS dataset is independent. Therefore, applying the Bonferroni correction usually gives us the most conservative p-value threshold. Because it is too conservative, it often leads to the generation of false negatives, and there may be few variants in the entire genome whose associated p-values can meet this standard. In this study, to investigate whether there are local genetic correlations between RA and MS, RA and IBD, and RA and T1D, we estimated the local genetic correlations between each pair of traits using ρ−HESS (37). The ρ−HESS method used Bonferroni correction as a threshold to identify the local genetic correlation between each pair of traits in the visualization results, so in the subsequent research analysis, we also used Bonferroni correction. Secondly, our results cannot be used to be representative of the global population or children, as the sample of RA, MS and IBD were taken from UK Biobank, individuals of European descent aged between 40 and 69 years, and the T1D summary statistics which individuals are European descent from the NHGRI-EBI GWAS Catalog. Thirdly, due to the lack of biological information at the individual level of genotype and phenotype datasets, we cannot determine whether the effect of polymorphic genes on disease risk is directed. Experimental studies are required to verify the pleiotropic genes identified in this study. Fourthly, existing gene annotation is not comprehensive, which leads to some SNPs not annotating genes. Fifthly, the vast majority of the risk SNPs and shared risk genes we identified are located in the MHC region on chromosome 6, although we used the clustering function of PLINK to identify independent and significant SNPs, due to the extensive linkage disequilibrium in the MHC region, it is possible that the risk SNPs and shared risk genes we identified are correlated. Furthermore, the functions of the newly identified shared risk genes are still unclear, and further studies are needed to understand the functions of the genes and their roles in pathophysiology. The function of the newly discovered shared risk genes is unclear, and there is still a long way to go to understand the function of genes and their role in the pathophysiology of disease. Finally, we did not analyze the combination of genetic and environmental factors that are known to influence autoimmune diseases, including smoking, diet, exercise, and medication. To sum up, further studies are needed to emphasize and explore the biological explanations, and efforts should be made to translate the findings into clinical research or practice. This study provides an effective theoretical basis for future research on the pathogenesis of autoimmune diseases, improvement of diagnostic methods and development of targeted therapies.

## 5 Conclusion

In this paper, strong genetic associations between RA and three autoimmune diseases have been explored. Through genetic estimation, it was found that there are local genetic correlation signals between RA and MS, RA and T1D. By cross-trait meta-analysis, it was found that there are independent genetic loci related to RA and MS, RA and IBD, RA and T1D. Based on gene correlation analysis, 82 common risk genes were found among the four diseases. Common risk genes are enriched in skin sun exposed lower leg, muscle skeletal, adipose subcutaneous, and 35 biological pathways. Through Mendelian randomization analysis, we found that there may be causal relationship between RA and T1D, RA and MS. Therefore, this study is helpful for the clinical treatment of RA, MS, IBD and T1D.

## Data availability statement

Publicly available datasets were analyzed in this study. The data can be found here: http://geneatlas.roslin.ed.ac.uk/ and https://www.ebi.ac.uk/gwas/.

## Author contributions

Y-PW contributed to the conception and design of the study, developed the method and wrote the manuscript. Z-GY gave the ideas and supervised the project, also revised the manuscript. All authors contributed to the article and approved the submitted version.
